# Control values of ovarian cancer tumor markers and standardisation of a protocol for sampling peritoneal fluid and performing washing during laparoscopy

**DOI:** 10.1186/1477-7819-12-278

**Published:** 2014-09-04

**Authors:** Katarina Galic Jerman, Borut Kobal, Marina Jakimovska, Ivan Verdenik, Katarina Cerne

**Affiliations:** Department of Gynecology, Division of Gynecology and Obstetrics, University Medical Centre Ljubljana, Zaloška 2, 1000 Ljubljana, Slovenia; Department of Gynecology and Obstetrics, Faculty of Medicine, University Ljubljana, Slajmarjeva 3, 1000 Ljubljana, Slovenia; Institute of Pharmacology and Experimental Toxicology, Faculty of Medicine, University Ljubljana, Korytkova 2, 1000 Ljubljana, Slovenia

**Keywords:** Ovarian cancer, Tumor markers, Peritoneal fluid, Peritoneal washing, Protocol standardization, sOPN, sCD44-v6, sVCAM-1, Flow cytometry

## Abstract

**Background:**

Determination of the tumor marker concentration in peritoneal fluid (PF) may help to assess its potential to detect small concentration changes between benign ovarian pathology and early stage ovarian cancer. Peritoneal washing, which can also be obtained when PF is absent, is already included in the International Federation of Gynecology and Obstetrics (FIGO) staging classification for ovarian cancer but sampling has not yet been standardized. Since our aim was to evaluate the relationship between marker concentration in PF and washing, standardization of the sampling protocol was a prerequisite to ensure reliable results.

**Methods:**

Thirty-three women with non-malignant pathology of the reproductive organs were included in the study. We used three promising tumor markers for evaluation of the marker concentration in local fluid: osteopontin (sOPN), splice variant 6 of sCD44 (sCD44-v6) and vascular cell adhesion molecule-1 (sVCAM-1). After aspiration of PF, washing of the uterus, ovaries and pelvic peritoneum was performed with saline solution. Patients were divided into two groups based on the solution volume: A-20 ml and B-50 ml. To determine the efficiency of washing in relation to solution volume, washing was repeated three times. Concentrations of markers in samples were determined using flow cytometry.

**Results:**

Mean concentrations of markers were significantly higher (*P* <0.001) in PF than in the first washing. We demonstrated a significant positive correlation between marker concentrations in PF and first washing (sOPN: *r* = 0.447, *P* = 0.048; sCD44-v6: *r* = 0.660, *P* = 0.002; sVCAM-1: *r* = 0.526, *P* = 0.017). When using a smaller solution volume for washing, significantly higher (sVCAM-1: 2.5-fold, *P* = 0.021; sOPN: 3-fold, *P* = 0.024) or equal (sCD44-v6) mean concentrations of tumor markers were obtained.

**Conclusions:**

Our work demonstrates for the first time that concentrations of sOPN, sCD44-v6 and sVCAM-1 in PF correlate with peritoneal washing in women with non-malignant pathology of the reproductive organs. This indicates that, for selected tumor markers, washing can replace PF when PF is absent. A standardized protocol for sampling PF and performing washing during laparoscopy was established.

## Background

Ovarian cancer usually presents in advanced stages, with a high mortality, but has a favorable prognosis if diagnosed at an early stage [1, 2]. The most clinically useful ovarian cancer blood tumor markers, cancer antigen 125 (CA125) and human epididymis protein 4 (HE4), are far from ideal for early detection of the disease due to the unacceptable level of specificity and sensitivity [2]. One of the challenges in this field is therefore to identify tumor markers for the detection of early-stage disease. However, it is questionable when the number of neoplastic cells is low, whether sufficient tumor product can reach the peripheral blood (a range of 0.1 to 20% of secreted protein is assumed) for early disease detection with diagnostic tests, taking into account the sensitivity of the blood assay [3]. We can therefore apply a different approach and evaluate the concentrations of ovarian cancer markers in the fluid of the local environment. This approach could help to elucidate the potential of new blood tumor markers for early-stage disease since changes in levels of tumor markers in local fluid, due to higher quantities, are more quickly detectable and are also more specific [4].

In addition to its use in tumor marker research, this approach has potential clinical applicability in patients with suspected adnexal masses, where determination of tumor markers not only in blood but also in local fluid, in combination with cytology, may be useful in order to distinguish more accurately between benign and malignant forms of ovarian neoplasm. However, to set a cut-off value for local fluid between benign and malignant disease, control values of tumor markers in women with benign pathology of the reproductive organs should first be determined.

In order to obtain control values of markers in local fluid, a sample of peritoneal fluid (PF) and/or peritoneal washing can be used, which is already incorporated in the International Federation of Gynecology and Obstetrics (FIGO) staging classification for ovarian cancer [5, 6] although the procedure has not yet been standardized. In early ovarian cancer, elevation of the concentration of tumor markers in the local environment may contribute to surgical staging, especially in cases in which PF is absent and a representative cytological sample is difficult to obtain [7–9]. Since peritoneal washing is used in such cases, knowing the relationship of tumor marker concentrations between PF and peritoneal washing is essential. However, a standard sampling protocol (SSP) is a prerequisite to ensure reliable results.

For a SSP, tumor markers are needed whose soluble forms are present in measurable concentrations in the PF of women with benign pathology of the reproductive organs. Osteopontin (sOPN), a splice variant 6 of CD44 (sCD44-v6) and vascular cell adhesion molecule-1 (sVCAM-1) were shown in our preliminary study [10] to be present in determinable concentrations in PF. In addition, all three are promising tumor markers present in ovarian cancer cells and/or mesothelial cells. They are involved in cell motility, adhesion and spreading free tumor cells through the peritoneal cavity [11–14]. Concentrations in samples, PF and/or peritoneal washing can be further compared to their serum concentrations, thereby offering many research and clinical applications if the correlation can be proved. As already demonstrated in advanced ovarian cancer patients, the results of sVCAM-1 concentrations in serum and ascites have shown a strong correlation, indicating that the serum values of sVCAM are the result of concentration changes in the local environment [14].

In present study we focused only on the proportion of concentrations of ovarian cancer tumor markers in PF and peritoneal washing using SSP, in order to elucidate whether washing could replace PF. SSP was performed in a group of patients with benign pathology of the reproductive organs using sOPN, sCD44-v6 and sVCAM-1. In particular, we tried to elucidate the influence of the solution volume used for performing washing on tumor marker concentrations in washing samples and on the efficacy of the washing procedure.

## Methods

### Patients

The study included 33 patients with benign pathology of the reproductive organs who were operated reject at the Department of Gynecology, University Medical Centre Ljubljana between December 2011 and September 2013. Exclusion criteria were gynecological malignancies, elevated parameters of inflammation (C-reactive protein and total white blood cell count), hysteroscopy and elevated standard tumor markers (Ca125, Ca15-3, Ca19-9 and CEA). Patients were in the reproductive (during follicular or luteal phases), perimenopausal or postmenopausal phase. Family, general, gynecological and obstetric history, indication for surgery, other relevant diseases and current therapy were collected from medical records. The purpose of the SSP was explained to all patients and written informed consent was obtained prior to enrolment. The study was approved by the Commission of the Republic of Slovenia for Medical Ethics (approval number: 82/01711) and in accordance with the Declaration of Helsinki.

### Protocol for aspirating peritoneal fluid and performing peritoneal washing during laparoscopy

During the procedure, patients underwent standard laparoscopy. No solution was injected before inflation that could alter the volume of PF. The SSP was not performed in the case of adhesions that would prevent its optimal execution. Immediately after entering the abdominal cavity, all the available PF was aspirated from the cavum Douglasi through the cannula into a syringe. This was followed by the washing procedure. If PF was not present, the washing procedure was performed directly after entering the abdominal cavity. Washing was performed by spilling 0.9% NaCl solution onto the surface of the uterus, ovaries and pelvic peritoneum. The solution was left for 2 minutes in the pelvic cavity. The total volume (in an ideal anatomical condition) was aspirated back into the syringe for calculating the absolute quantity of tumor markers.

Based on the solution volume for washing, patients were divided into two groups: group A (smaller volume) and group B (larger volume). We took care to include patients with various diagnoses in each group. Because the procedure was time-demanding, washing was initially performed on only seven patients in each group and their samples were analyzed in order to choose which volume was better for final standardization of the washing procedure on a larger number of patients. First washing in group A and group B was performed with 20 and 50 ml, respectively. To determine the efficiency of washing in relation to the solution volume, the procedure was repeated twice after the first washing; in group A by using 20 ml and 10 ml of solution and in group B by using 50 ml and 20 ml of solution. The last washing was performed with a smaller volume to avoid the concentration of tumor markers falling below the detection limit for the analytical method. After comparison of tumor marker concentrations between the two groups, we selected the smaller volume and included an additional 19 patients. Since the emphasis of our analysis was on the first washing, which is clinically useful, we performed it on all additional 19 patients (altogether on 26 patients); the second and third washings were performed on 13 additional patients (altogether on 20 patients).

Samples of PT and washings were immediately transferred into a conical tube, which was kept on ice until centrifugation at 1000 × g for 10 minutes at 4°C within 30 minutes. The volume of each PF and washing sample was recorded before centrifugation. Samples contaminated with blood were excluded from the analysis. Supernatants were stored in aliquots at -80°C. No more than two freeze-thaw cycles were allowed for any sample.

### Analysis of sOPN, sCD44-v6 and sVCAM-1 with bead-based flow cytometric assay

Concentrations of sOPN, sCD44-v6 and sVCAM-1 in samples were measured separately using a FlowCytomix Simplex Kit (eBioscience, Vienna, Austria). The kit consists of fluorescent microspheres with an emission wavelength at 700 nm (5 μm diameter for sOPN and sCD44-v6 analysis and 4 μm diameter for sVCAM-1 analysis). Microspheres are coated with specific antibodies raised against each of the analytes (sOPN, sCD44-v6 or sVCAM-1). They also contain a biotin-conjugated second antibody and streptavidin-phycoerythrin emitting at 575 nm. Samples were run on a Cell Lab Quanta™ SC-MPL (Beckman Coulter, Fullerton, United States). Electronic volume versus side scatter gating was employed to exclude any sample particles other than 5 μm (4 μm) microspheres. Samples were acquired by Cell Lab Quanta^TM^ SC-MPL software (Beckman Coulter, Fullerton, United States) and analyzed using Flowcytomix™ Pro 3.0 software (eBioscience, Vienna, Austria). The lower limits of detection of sOPN, sCD44-v6 and sVCAM-1 were 0.432 ng/ml, 0.126 ng/ml and 0.9 ng/ml, respectively.

### Statistical analysis

To determine the efficiency of washing in relation to the volume of solution for performing the procedure, we calculated the absolute quantity of sOPN, sCD44-v6 and sVCAM-1 separately in three consecutively performed washings. In the calculation, the tumor marker concentration (ng/ml) in each sample of washing was multiplied by the volume of aspired washing (ml). We summed the absolute quantities of tumor marker in all three consecutively performed washings to determine the percentage of each tumor marker acquired from the first washing. All data are presented as mean ± standard error of mean (SEM). Pearson’s correlation coefficient was used to calculate the strength of the relationship between normally distributed variables. Data were compared by the Student’s unpaired t-test. A *P* value of <0.05 was considered significant. Statistical analysis was performed using software statistical package SPSS, version 19 (IBM Statistics, New York United States).

## Results

The characteristics of the investigated 33 aprove patients are summarized in Table 1.

**Table 1:** **Patient characteristics**

Parameters	
Number of patients	33
age (average ± SEM)	43 ± 1.82 years
range	21-69 years
**Diagnosis, number of patients (%)**	
benign ovarian cyst	8 (24%)
myoma of the uterus	16 (48%)
pelvic pain, sterilization	7 (24%)
preventive adnexectomy	2 (6%)
**Period, number of patients (%)**	
reproductive	23 (70%)
perimenopausal	6 (18%)
Postmenopausal	4 (12%)
**Phase of menstrual cycle, number of patients (%)**	
luteal	10 (30%)
follicular	7 (21%)
not applicable	16 (48%)
**Free peritoneal fluid**	
present, number of patients (%)	26 (79%)
absent, number of patients (%)	7 (21%)
volume (mean ± SEM)	6.03 ± 1.22 ml
**Contraception, number of patients (%)**	
intrauterine device	4 (12%)
combined hormone therapy	3 (9%)

### The influence of solution volume used for washing on sOPN, sCD44-v6 and sVCAM-1 concentrations in the washing samples and on the efficiency of the washing procedure

**Figure 1:**
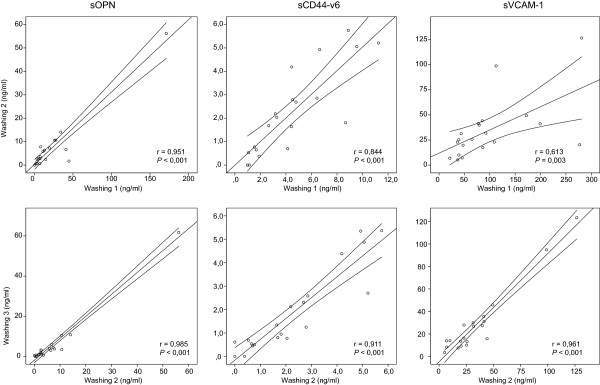
**Correlation of sOPN, sCD44-v6 and sVCAM-1 concentrations between first and second washings, as well as between second and third washings.** Three washings were performed consecutively in patients of group A with 20 ml, 20 ml and 10 ml of 0.9% NaCl solution.

Based on the volume of solution used for washing, the patients were divided into two groups: group A (smaller volume) and group B (larger volume). The emphasis of our analysis was on the first washing, which can be used as a substitute for PF if PF is not present. In the first washing of patients in group A, the mean sVCAM-1 concentration was 2.5-fold higher (*P* = 0.021) and sOPN 3-fold higher (*P* = 0.024) than the mean concentration in the first washing of patients in group B. However, there was no effect of solution volume on mean sCD44-v6 concentration in the first washing (Table 2). In both groups, the mean concentrations of sOPN, sCD44-v6 and sVCAM-1 in three consecutively performed washings decreased, except the sVCAM-1 concentration between the second and third washings in group B (Table 2). A significant positive correlation between the first and second washings was observed for sOPN (*r* = 0.951, *P <*0.001), sCD44-v6 (*r* = 0.884, *P <*0.001) and sVCAM-1 (*r* = 0.813, *P* = 0.003), as well as between the second and third washings for sOPN (*r* = 0.985, *P <*0.001), sCD44-v6 (*r* = 0.991, *P <*0.001) and sVCAM-1 (*r* = 0.961, *P <*0.001) (Figure 1).

**Table 2:** **Concentrations of sOPN, sCD44-v6 and sVCAM-1 (average ± SEM) in peritoneal fluid and in washings performed with 0.9**% **NaCl solution**

Sample (number of patients)	sOPN	sCD44-v6	sVCAM-1
		**(ng/ml)**	
**Groups A + B** (33)			
Peritoneal fluid (26)	132.1 ± 22.9	45.3 ± 3.4	438.8 ± 11.7
range	21.4 - 483.1	19.3 - 89.3	311.8 - 588.43
**Group A** (smaller volume of solution for performing washing)			
Washing 1A (26)	20.0 ± 6.5	4.6 ± 0.5	108.2 ± 14.7
Washing 2A (20)	7.2 ± 2.7	2.3 ± 0.4	34.4 ± 6.7
Washing 3A (20)	6.0 ± 3.0	1.8 ± 0.4	28.3 ± 6.7
**Group B** (larger volume of solution for performing washing)			
Washing 1B (7)	6.7 ± 1.7	5.5 ± 1.3	46.2 ± 11.6
Washing 2B (7)	3.0 ± 1.8	2.6 ± 0.9	20.7 ± 6.7
Washing 3B (7)	2.2 ± 0.5	2.1 ± 1.0	22.1 ± 8.5
**Concentration ratio**			
Peritoneal fluid : washing 1A	6.6	9.8	4.1

Three washings were performed consecutively in patients of group A with a smaller volume of solution (1A: 20 ml, 2A: 20ml and 3A: 10 ml) and in patients of group B with a larger volume of solution (1B: 50 ml, 2B: 50 ml and 3B: 20 ml).

**Figure 2:**
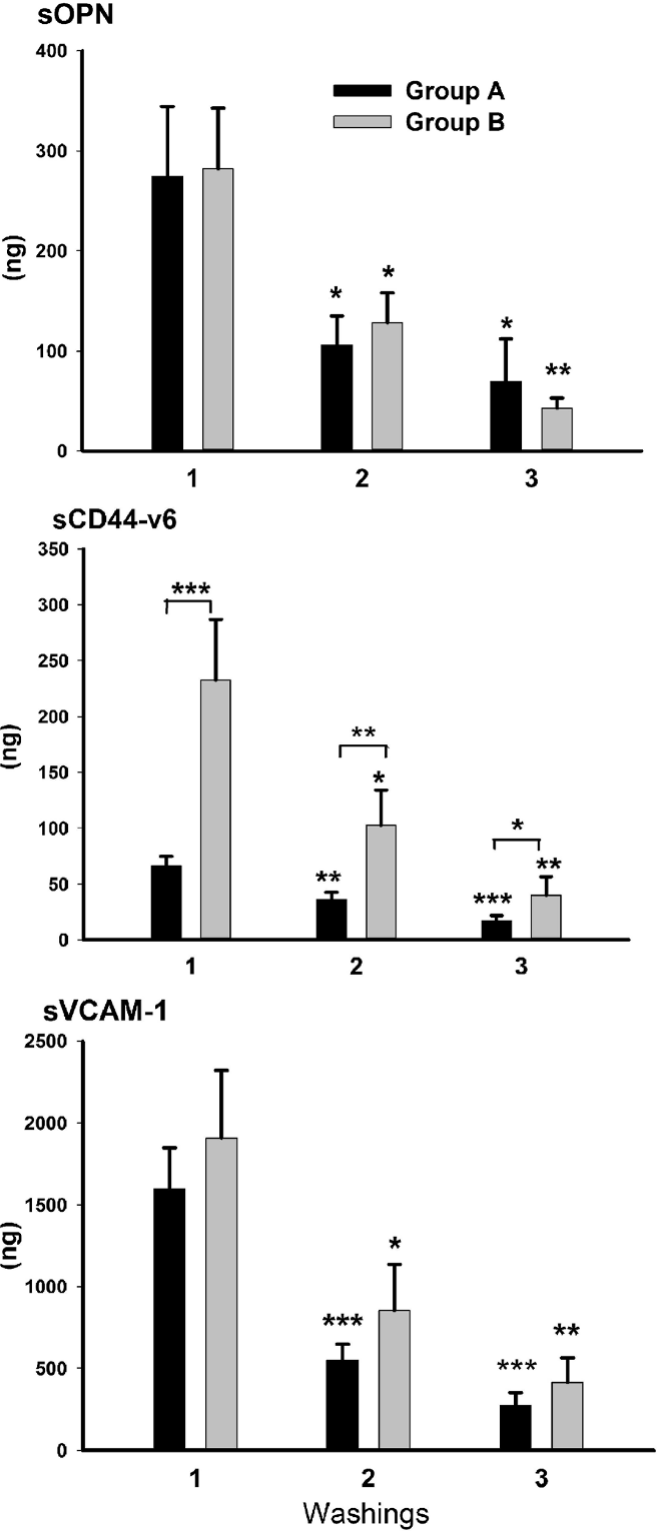
**Comparison of absolute quantity (ng) of sOPN, sCD44-v6 and sVCAM-1 among three consecutively performed washings, as well as between group A (black bars) and group B (grey bars).** In comparison to the first, the absolute quantity of all three tumor markers significantly decreased in the second and third washings within both groups. There was no significant difference in absolute quantity of sOPN and sVCAM-1 between groups A and B in any of the three washings. However, there was a significant difference in absolute quantity of sCD44-v6 between groups A and B in all three washings. Washings were performed consecutively in patients of group A with a smaller volume of solution (1A: 20 ml, 2A: 20 ml and 3A: 10 ml) and in patients of group B with a larger volume of solution (1B: 50 ml, 2B: 50 ml and 3B: 20 ml). Tumor marker absolute quantity was calculated by multiplying tumor marker concentration (ng/ml) with the volume of aspirated washing (ml). *P <0.05, **P <0.01, ***P <0.001.

In order to elucidate the influence of volume size on the efficiency of the washing procedure, we calculated the absolute quantity of sOPN, sCD44-v6 and sVCAM-1 in three consecutively performed washings. For this purpose, we measured the volumes of aspirated washings. In group A, the mean aspirated volume of the first, second and third washings were 15 ml, 16 ml and 9 ml and in group B were 43 ml, 42 ml and 19 ml, respectively. The proportions of aspirated washing solution based on the solution volume used for washing were 73%, 82% and 90% in group A and 85%, 85% and 95% in group B. The mean absolute quantity of sOPN and sVCAM-1 in all three washings of patients in group A did not significantly differ from that in group B. In contrast, the mean absolute quantity of sCD44-v6 of all three washings of patients was significantly higher in group B than in group A; first washing: *P <*0.001, second washing: *P* = 0.002 and third washing: *P* = 0.036 (Figure 2). In three consecutively performed washings, the mean absolute quantity of sOPN, sCD44-v6 and sVCAM-1 decreased in patients of both groups. In the second and third washings of both groups, the mean concentrations of sOPN, sCD44-v6 and sVCAM-1 were significantly lower than in the first washing (Figure 2). With the first washing, we acquired 61 to 65% of the total quantity of each tumor biomarker in the three consecutively performed washings, regardless of solution volume.

### Concentrations of sOPN, sCD44-v6 in sVCAM-1 in peritoneal fluid and the first washing of patients in group A

**Figure 3:**
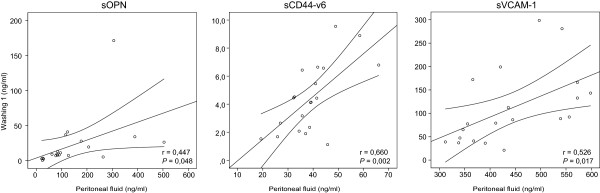
**Correlation of sOPN, sCD44-v6 and sVCAM-1 concentrations between peritoneal fluid and the first washing in patients of group A.** Washing was performed with 20 ml of 0.9% NaCl solution.

Mean concentrations of tumor markers in PF (sOPN: 144.42 ± 29.07 ng/ml, sCD44-v6: 39.20 ± 2.37 ng/ml and sVCAM-1: 441.78 ± 20.79 ng/ml) were significantly higher (*P* <0.001) than in the first washing (sOPN: 19.98 ± 6.57 ng/ml, sCD44-v6: 4.56 ± 0.56 ng/ml and sVCAM-1: 108.21 ± 14.75 ng/ml) (Table 2). To elucidate whether the first washing might replace PF if PF is not present, we evaluated the association of tumor marker concentrations in PF with their concentrations in the first washing. We determined a significant positive correlation between concentrations of sOPN (*r* = 0.447, *P* = 0.048), sCD44-v6 (*r* = 0.660, *P* = 0.002) and sVCAM-1 (*r* = 0.526, *P* = 0.017) in PF and the first washing (Figure 3).

### Clinical characteristics of the investigated patients in relation to the concentration of sOPN, sCD44-v6 and VCAM-1

Concentrations of sOPN, sCD44-v6 and sVCAM-1 in PF or washing samples were not associated with the presence of benign ovarian cysts, phase of menstrual cycle or contraception. The mean volume of PF was 7.66 ± 1.38 ml (group A: 7.26 ± 1.54 ml; group B: 8.98 ± 3.3 ml). PF was present in 26 (79%) patients. However, the presence of PF was not associated with a higher concentration of tumor markers in the first washing.

## Discussion

Extensive research in the field of serum tumor markers for early detection of ovarian cancer is currently underway, with many promising candidates, although none of them have as yet fulfilled expectations [15, 16]. The aim of this study was to apply a different approach and evaluate the control concentrations of ovarian cancer markers in local fluid of patients with benign pathology of the reproductive organs. This approach could help to elucidate the potential of promising blood tumor markers for early-stage disease, since tumor markers closer to the origin of disease are more specific and their changes of concentration can be detected faster. We used PF and/or peritoneal washing to obtain control values of tumor markers in local fluid, which is already included in the FIGO staging classification for ovarian cancer although the protocol of sampling has not so far been standardized [5, 6]. In early stages of ovarian cancer, when ascites is often not present, PF accumulated in the cavum Douglasi plays a crucial role in disease progression as a result of dissemination of detached tumor cells into the peritoneal cavity and adhesion to mesothelial cells of the peritoneum and other abdominal organs [17–19].

That SSP is a prerequisite to ensuring reliable results was also demonstrated by our preliminary results for sVCAM-1 in patients with a benign pathology of reproductive organs, in which samples were collected without SSP, with the exception of the instruction to perform washing with 20 ml of 0.9% NaCl solution. sVCAM-1 has a wide concentration range and concentrations of sVCAM-1 in some samples were below the detection limit for analytical assay (unpublished observations KC, BK, MJ). In order to elucidate whether the findings of the present study can be generally applied for tumor markers and not just for sVCAM-1, we included sOPN and sCD44-v6 in our study. sOPN and sCD44-v6 were shown in our preliminary study [10] to be present in determinable concentrations in PF. sOPN and sVCAM-1 are included in the list of top blood tumor markers for early detection of ovarian cancer [15, 16] and sCD44-v6 has been shown to be useful in differential diagnosis of benign and malignant ascites [13]. Ascites is a pathological increase in the volume of PF present in 27% of patients with early-stage ovarian cancer and in 50% of borderline ovarian cancers [8].

Performing washing is a complex procedure involving many factors, which is why each surgeon must follow the instructions of the SSP if comparable results among patients are to be obtained. The current work therefore standardized the main factors of the sampling procedure: determination of the solution volume and the time the solution is left in the pelvic cavity, specification of areas for washing and accuracy during aspiration of the whole solution volume (in an ideal anatomical condition) back into the syringe. The solution volume is the most important factor in determining the tumor marker concentration. We found very varied data on the volume in published studies, from 10 to 1000 ml [7, 9, 20, 21]. In order to elucidate the influence of solution volume on tumor marker concentrations, we compared the results acquired with two different volumes (20 in 50 ml), while the time and areas of washing were always the same. The mean concentration of sOPN was 3-fold and the mean concentration of sVCAM-1 was 2.5-fold higher when washing with a smaller volume was performed, while the mean concentrations of sCD44-v6 were similar. These results demonstrate that a smaller volume is more appropriate for washing, in order to avoid the concentration of tumor marker falling below the detection limit for the analytical method and, consequently, a decrease in tumor marker sensitivity. Additionally, the procedure was technically easier to perform because a smaller volume of washing sample can be aspirated back into the syringe faster than a larger one. In contrast to our preliminary results for sVCAM-1 (unpublished observations KC, BK, MJ), none of the results when using SSP were below the detection limit. When we calculated the absolute quantity of sOPN and sVCAM-1, we found that the larger volume only diluted the samples. The amounts of the tumor markers were the same regardless of volume, so the effectiveness of washing was also the same. However, the absolute quantity of sCD44-v6 was higher when washing was performed with the larger volume. We discovered that, probably due to specific physical factors, the baseline values of sCD44-v6 in group B were different from those in group A, since the mean concentration of sCD44-v6 in the PF of group B patients was statistically significantly higher than that in group A, whereas the mean concentrations of sOPN and sVCAM-1 in PF were not significantly different between groups. Another explanation might be the different physiochemical properties of the sCD44-v6 molecule (such as better solubility in saline solution). A limitation of our study was that it was impossible to perform the washing procedure with different volumes on the same patient. In an attempt to clarify the process of washing the pelvic cavity, we repeated the same procedure twice and our results demonstrated that we had fully washed the tumor markers from the pelvic surface. First, using the absolute quantity, we demonstrated that the mean absolute quantity of tumor markers in three consecutively performed washings decreased. Second, when tumor marker concentrations were compared among three consecutively performed washings, their concentrations were strongly correlated between the first and second washings, as well as between the second and third washings.

PF is an excellent body fluid for determining control values of tumor markers in the local tumor environment, although it is known that PF is sometimes not present in the cavum Douglasi [7, 22]. Comparable to published data, we found PF was absent in 21% of patients [7]. In such a situation, washing can replace PF as a control of the local environment. To discover whether this is possible, concentrations of tumor markers were determined separately in PF and washing. In patients with PF present, therefore, we first aspirated all fluid from the cavum Douglasi and then performed washing. As mentioned earlier, a smaller volume of solution is appropriate for washing, so we further evaluated only the results of tumor marker concentrations in washing obtained with the smaller volume. The results of the present study determined a positive correlation between PF and washing concentrations of tumor markers, showing that washing can replace PF in a situation in which PF is absent. The mean concentrations of tumor markers were significantly higher in PF than in washing; approximately 7-fold for sOPN, 10-fold for sCD44-v6 and 4-fold for sVCAM-1. These results demonstrate the importance of ensuring that all PF is first aspirated from the cavum Douglasi. We evaluated whether higher levels of tumor markers in washing are associated with the presence of PF and found that the presence of PF was not associated with higher concentrations in washing. This finding also proved that our results of washings were not affected by inaccurate aspiration of PF. The results of the sVCAM-1 concentration in PF and washing in this study showed that the wide range of concentrations in our preliminary results of sVCAM-1 in washings (unpublished observations KC, BK, MJ) was probably because of sampling without a standardized protocol. Especially for sVCAM-1 in PF, the concentration range was much narrower (10-fold) when using the SSP. A consequence of the wide concentration range is a large standard deviation, which affects setting the cut-off value for the tumor marker [3]. We also determined preliminary control values of sOPN, sCD44-v6 and sVCAM-1 in PF and washing based on the SSP, although a follow-up study with a larger number of patients is needed to determine their final values.

The SSP could also be applied in patients with ovarian cancer. Peritoneal washing for cytology is already part of routine practice in ovarian cancer, either during initial evaluation or second-look procedures as part of the FIGO staging classification, being of the utmost importance in early-stage disease for detecting early spread of the disease [5, 6]. Since ascites is not present in 83% of patients with early-stage ovarian cancer and in 50% of patients with borderline tumors [8], washing provides a sample of local fluid that can be obtained in such cases. Among factors that may contribute to the relatively high false-negative rate (20%) of cytology is infrequent exfoliation of malignant cells, but washing not being performed correctly can also be a factor [23, 24]. A SSP can thus make a contribution to cytology sampling, since an increased level of tumor marker(s) in peritoneal washing could still be detected [9]. Determination of tumor marker(s) concentration in washing may thus be useful in combination with cytology in order to obtain more accurate results, especially in classification of early-stage disease.

The next step in our research will be to evaluate the relationship between local concentrations and those in serum. According to the results of sVCAM-1 obtained in advanced cancer patients [14], serum values in benign conditions, as well as in ovarian cancer, should demonstrate the same ratio of concentrations towards tumor marker concentrations in the local environment. To evaluate the potential of a novel tumour marker to discriminate between benign and malignant disease, its level in peritoneal washing of patients with benign pathology of the reproductive organs as well as follow up study on sufficient number of patients with stage I ovarian cancer should be conducted where SPSS will be applied.

## Conclusions

This is the first study to demonstrate a positive correlation between concentrations of selected tumor markers (sOPN, sCD44-v6 and sVCAM-1) in PF and washing in women with non-malignant pathology of the reproductive organs, which indicates that for selected tumor markers, washing can replace PF when PF is absent. The present study shows that a SSP is necessary to obtain comparable results among patients. The SSP could also be applied in patients with ovarian cancer. We standardized the main factors of the sampling procedure, in particular the solution volume for performing washing. A smaller volume of washing solution is preferable to a larger one because of higher or equal concentrations of markers in samples, which allow their detection without loss of efficiency of washing, and the procedure is also technically easier to perform.

## Abbreviations

FIGO: International Federation of Gynecology and Obstetrics
PF: Peritoneal fluid
sCD44-v6: Splice variant 6 of sCD44
sOPN: Osteopontin
SSP: Standardized sampling protocol
sVCAM-1: Vascular cell adhesion molecule-1.
